# Avascular osteonecrosis mechanism – between osteoporosis and antiphospholipid syndrome

**DOI:** 10.1186/1471-2334-14-S4-P27

**Published:** 2014-05-29

**Authors:** Anca Ruxandra Negru, Cristina Popescu, Alina Lobodan, Raluca Dulamă, Irina Lăpădat, Mihaela Rădulescu, Victoria Aramă

**Affiliations:** 1 National Institute for Infectious Diseases “Prof. Dr. Matei Balş”, Bucharest, Romania

## 

HIV infected patients receiving antiretroviral therapy (ART) can develop avascular osteonecrosis, 45 times greater than the general population. Avascular osteonecrosis increased in the last few years in patients with HIV infection. The most important mechanisms for avascular osteonecrosis in HIV-infected patients are: changes in bone metabolism, especially osteoporosis correlated with protease inhibitors and coagulopathy and antiphospholipid syndrome, frequently described in HIV infection. Aim: to describe different mechanisms of avascular osteonecrosis

We present two HIV-infected patients under antiretroviral therapy who developed avascular osteonecrosis. First patient, male, 38 year-old, with AIDS-C3, with good immuno-virological outcome under AZT-3TC and lopinavir/ritonavir, developed severe osteoporosis after 5 years of ART, diagnosed by DXA test with a T-score <-2,5. The patient had: C4-C5-C6 severe osteoporosis with high fracture risk, with segmental instrumentation applied at those levels, and then bilateral avascular necrosis of femoral head (ANFH) with bilateral hip arthroplasty. ART regimen was changed and the patient received 3TC-ABC and Nevirapine. The patient didn’t have other risk factors for avascular osteonecrosis or osteoporosis: nonsmoker, normal CD4, undetectable viral load, without dyslipidemia. Despite ART changes, the patient developed bilateral osteonecrosis of the right knee and of both humeral heads.

The second patient, male, 23 year-old with HIV-B3, was treated in 2007 with AZT-3TC and lopinavir/ritonavir. After 9 months of ART the patient had normal CD4 count and undetectable viral load but developed right ANFH stage II, diagnosed by MRI, without surgery recommendation. DXA didn’t show signs of osteoporosis or osteopenia. The ART regimen was changed and the patient received 3TC-ABC and raltegravir with good ANFH outcome. After one year the patient discontinued the ART. One year later symptoms related with avascular osteonecrosis reappeared and bilateral ANFH was diagnosed by MRI. The patient had low CD4 count (<200/cmm), high HIV viral load and positive antiphospholipid antibodies. The patient had risk factors for coagulopathies: smoker, recreational drug user, alcohol consumer, uncontrolled HIV infection with low CD4. We restarted the same regimen with 3TC-ABC and raltegravir with a good outcome for bone affliction. When HIV infection was well-controlled, the antiphospholipid antibodies became negative and bone affliction was improved.

We emphasize the importance of metabolic disturbances in HIV-infected patients, among them avascular osteonecrosis with different mechanisms. Both, ART and uncontrolled HIV infection can affect bone metabolism and vascularization.

